# Supplementary material for: AgRP neuron activity enhances reward-related consummatory behaviors during energy deficit in mice
Source: Commun Biol. 2025 Aug 4;8:1152. doi: 10.1038/s42003-025-08620-9 (PMC12321993; doi:10.1038/s42003-025-08620-9)
Supplement: Supplementary file 2 — Supplementary Information [file 42003_2025_8620_MOESM2_ESM.pdf]

Supplementary Figure 1.

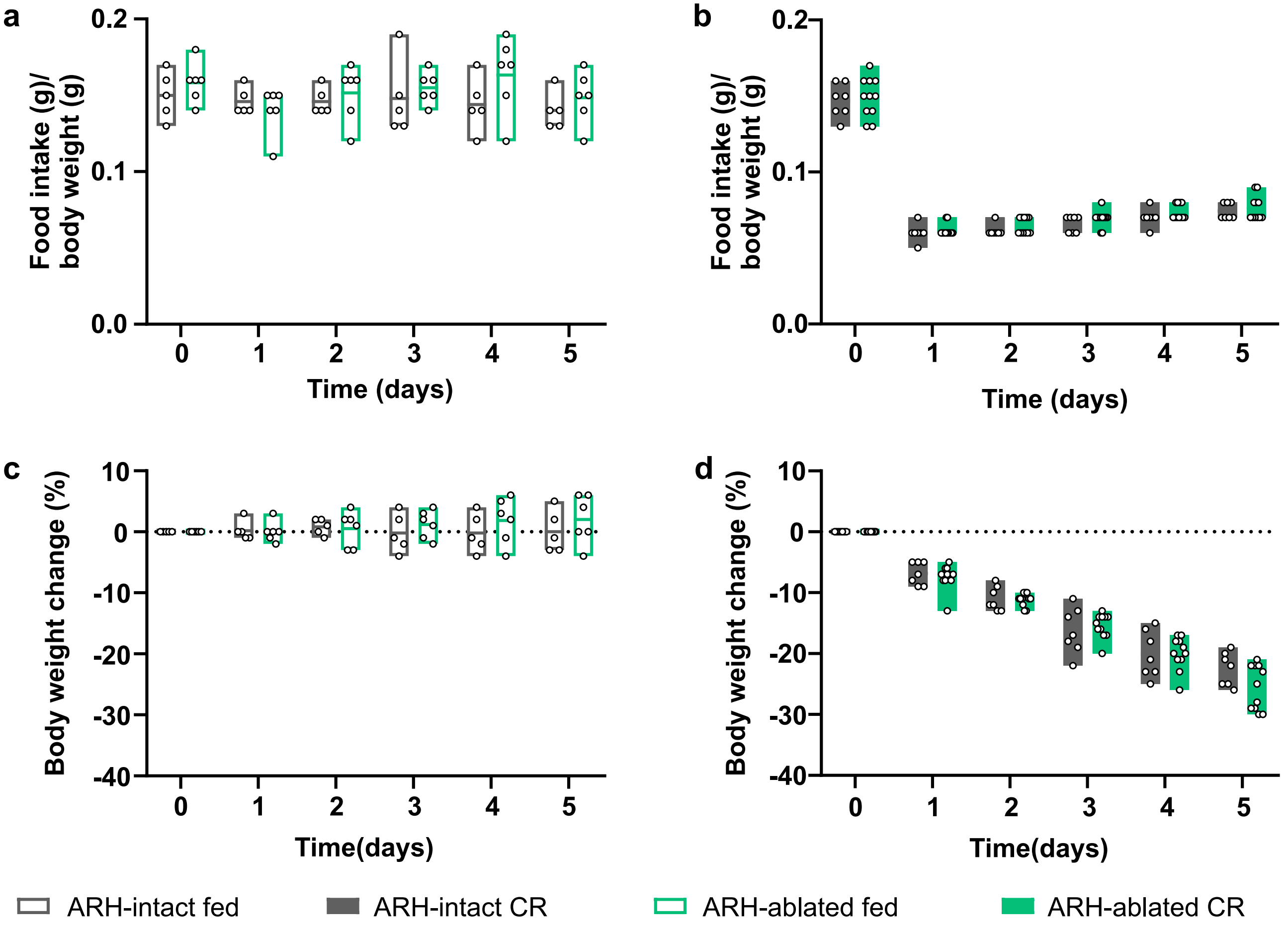

Supplementary Figure 1. ARH-intact and ARH-ablated calorie-restricted (CR) mice show similar food intake and body weight changes. (a, b) Boxplots of food intake normalized to body weight in *ad libitum*-fed (**a**, *p*-treatment × time=0.504; *p*-time=0.314; *p*-treatment=0.288) and CR (**b**, *p*-treatment × time=0.991; *p*-time<0.001, Cohen's *f*=6.65; *p*-treatment=0.320) mice with either intact or ablated ARH (n=5–6 mice per group). **(c, d)** Boxplots of percent body weight change in *ad libitum*-fed (**c**, *p*-treatment × time=0.318; *p*-time=0.685; *p*-treatment=0.551) and CR (**d**, *p*-treatment × time=0.079; *p*-time<0.001, Cohen's *f*=4.79; *p*-treatment=0.584) mice with intact or ablated ARH (n=7–11 mice per group). Data are presented as mean±SEM; error bars indicate SEM. Data was analyzed using two-way ANOVA.

Supplementary Figure 2.

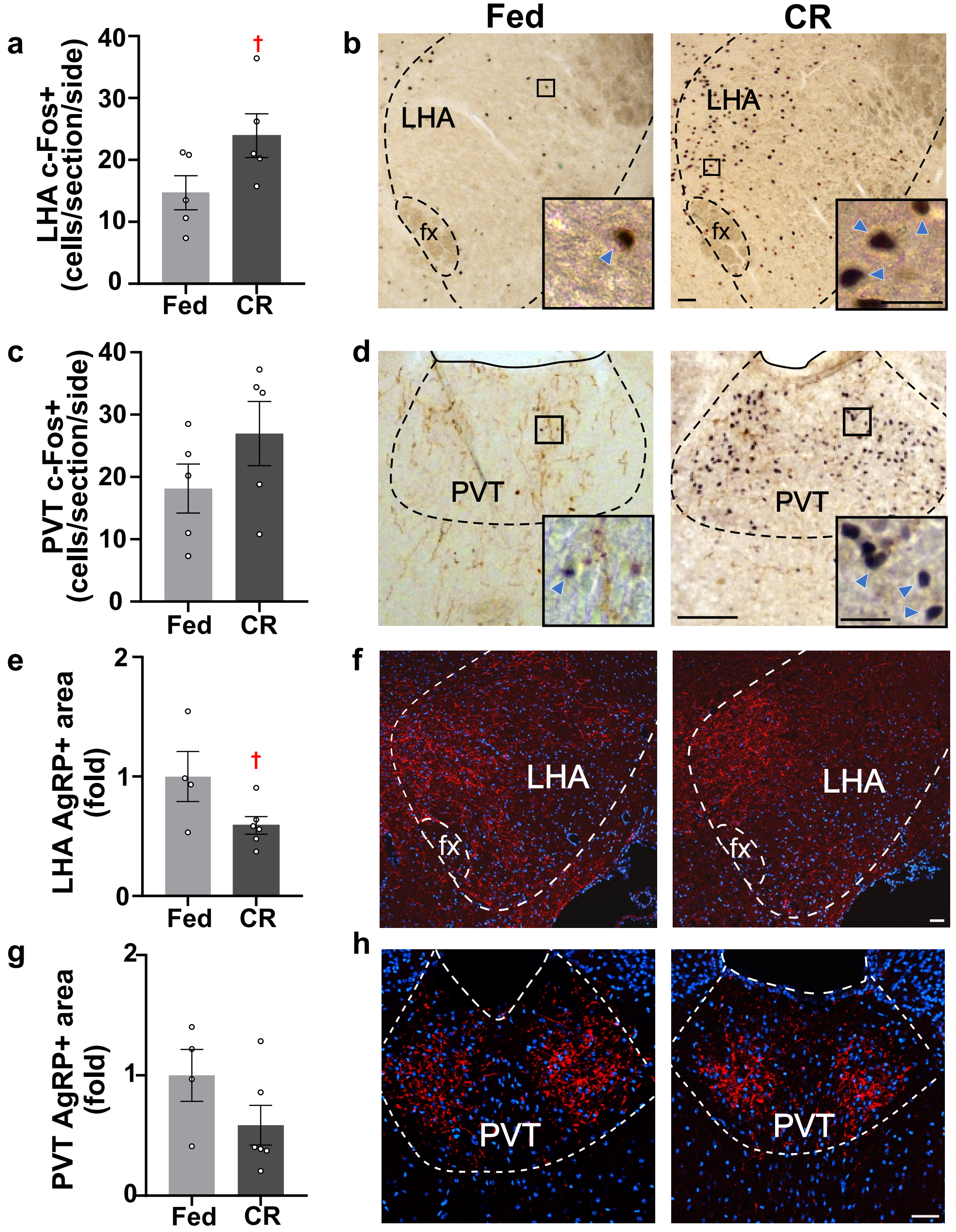

Supplementary Figure 2. Calorie restriction does not induce c-Fos expression in all AgRP neuronal targets. (a, c) Bar graphs showing the number of c-Fos+ cells in the lateral hypothalamic area (LHA; *p*=0.074) and the paraventricular thalamic nucleus (PVT; *p*=0.211), respectively, in *ad libitum*-fed (n=5) or calorie restricted (CR) (n=5) wild-type (WT) mice. **(b, d)** Representative photomicrographs of coronal sections through the LHA and PVT, respectively, processed with chromogenic immunohistochemistry for c-Fos (brown signal; blue arrowheads). **(e, g)** Bar graphs showing the quantification of AgRP-immunoreactive fluorescent area in the LHA (*p*=0.066) and PVT (*p*=0.161), respectively, in *ad libitum*-fed WT mice (n=4) or CR mice (n=6). **(f, h)** Representative fluorescent photomicrographs of coronal sections through the LHA and PVT, respectively, showing AgRP immunoreactivity (red). Scale bars: 50 μm (low magnification), 25 μm (high magnification). Data are presented as mean±SEM; error bars indicate SEM. Comparisons were made using unpaired t-tests. † p<0.1 vs. ad libitum-fed mice. fx, fornix.

Supplementary Figure 3.

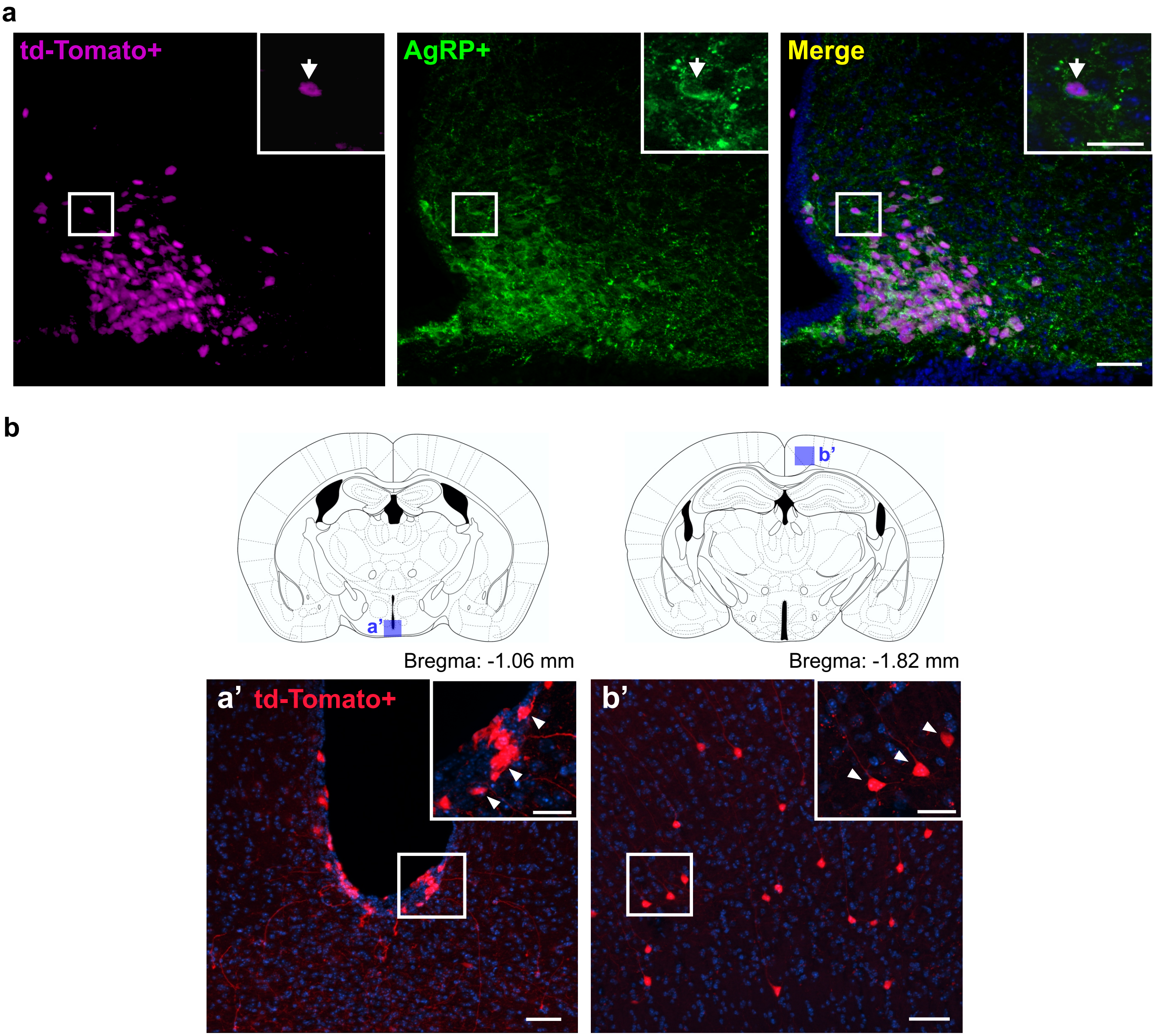

Supplementary Figure 3. AgRP-Cre mice show Cre expression in AgRP neurons of the hypothalamic arcuate nucleus (ARH) and in other brain areas. Representative photomicrographs of coronal brain sections from AgRP-Cre reporter mice expressing td-Tomato in Cre-expressing neurons. Mice were intracerebroventricularly injected with colchicine and subjected to fluorescent immunohistochemistry against AgRP. **a**) Photomicrograph showing the ARH, where td-Tomato+ (magenta) neurons co-express AgRP (green). **b**) Other regions displaying td-Tomato+ (red) neurons without AgRP immunoreactivity include (**a'**) the ependymal layer and (**b'**) the cortex. Scale Bar: 50 μm (low magnification) and 25 μm (high magnification).

Supplementary Figure 4.

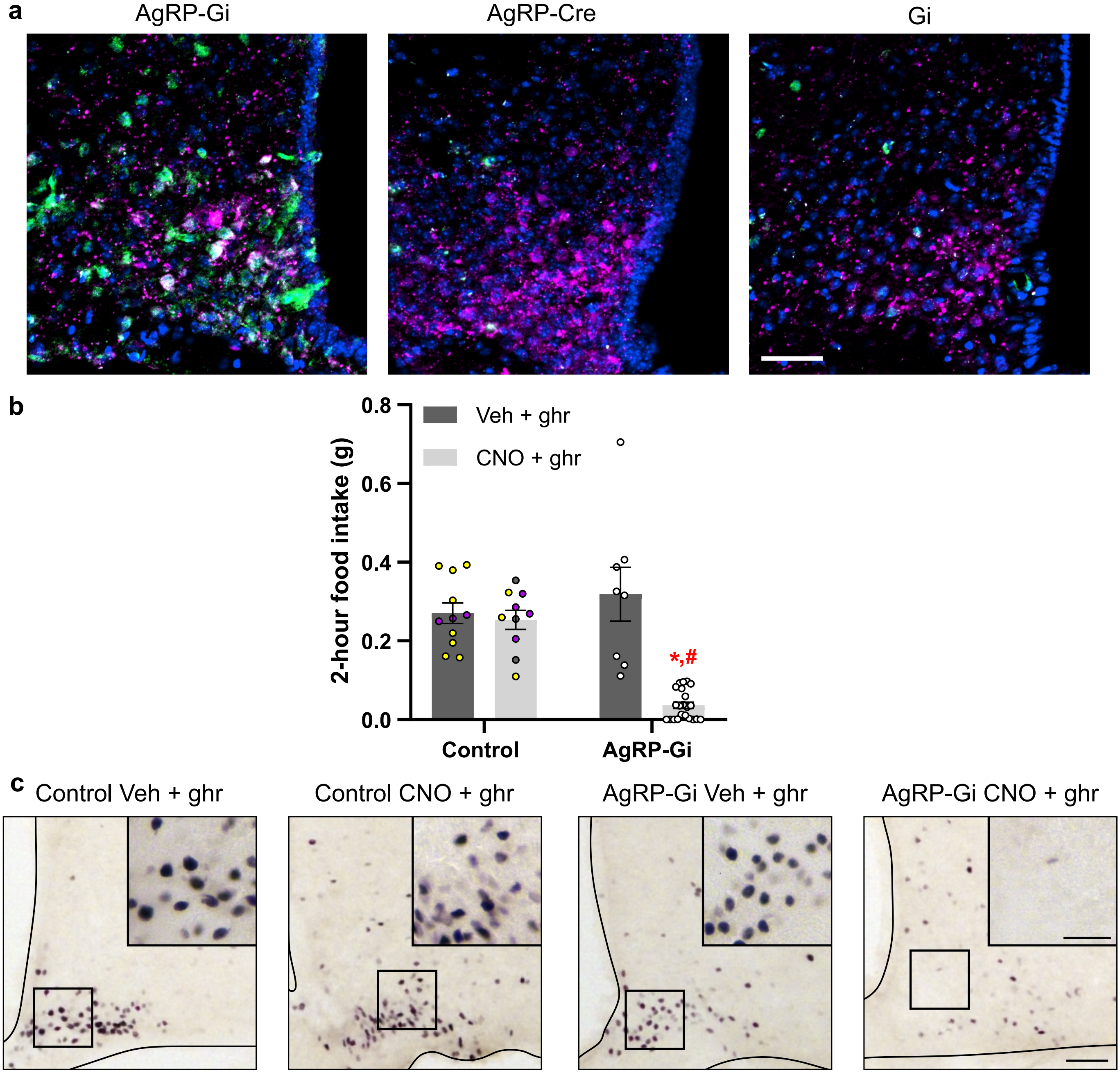

Supplementary Figure 4. CNO treatment inhibits ghrelin-induced food intake in AgRP-Gi mice. (a) Representative photomicrographs of the arcuate nucleus of the hypothalamus (ARH) from coronal brain sections of AgRP-Gi, AgRP-Cre, and Gi mice, subjected to immunohistochemistry for eGFP (green) and AgRP (magenta). **(b)** Bar graph showing 2-hour food intake in control mice (wild-type [WT]: grey; AgRP-Cre: yellow; Gi: purple) and AgRP-Gi mice following vehicle (n=11 and 8 per group) or CNO (n=10 and 22 per group) and ghrelin treatment. Two-way ANOVA: *p*-treatment × genotype<0.001, Cohen's *f*=0.68. Tukey's post hoc test: Vehicle-treated control vs. CNO-treated control, *p*=0.977; Vehicle-treated control vs. Vehicle-treated AgRP-Gi, *p*=0.689; CNO-treated control vs. CNO-treated AgRP-Gi, *p*<0.001; and Vehicle-treated AgRP-Gi vs. CNO-treated AgRP-Gi, *p*<0.001. Data are presented as mean±SEM; error bars represent SEM. Data were analyzed by two-way ANOVA. *, p<0.05 vs. same genotype, different treatment; #, p<0.05 vs. different genotype, same treatment. **(c)** Representative photomicrographs of coronal ARH sections from control and AgRP-Gi mice treated with vehicle or CNO and then with ghrelin, subjected to chromogenic immunohistochemistry for c-Fos (purple). Scale bars: 50 μm (low magnification) and 25 μm (high magnification).

Supplementary Figure 5.

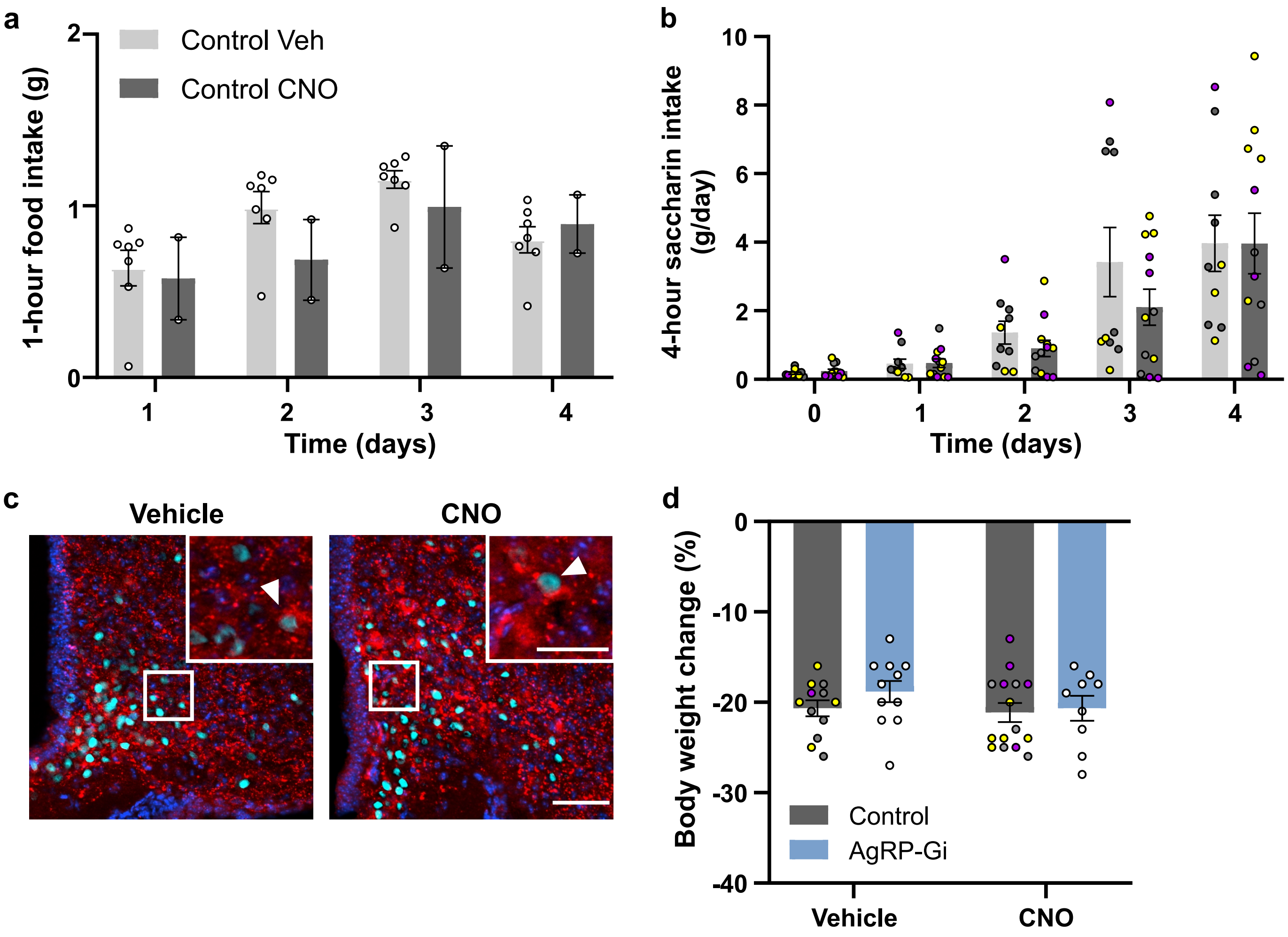

Supplementary Figure 5. CNO treatment does not induce any effect in calorie-restricted (CR) control mice. (a) Bar graph showing 1-hour food intake after saccharin exposure in vehicle- (n=7) or CNO-treated (n=2) control mice. Two-way ANOVA: *p*-treatment × time=0.293; *p*-time=0.002, Cohen's *f*=0.99; *p*-treatment=0.501. **(b)** Saccharin intake in CR control mice (wild-type [WT]: grey; AgRP-Cre: yellow; Gi: purple) treated with vehicle (n=10) or CNO (n=12) prior to saccharin exposure. Two-way ANOVA: *p*-treatment × time=0.436; *p*-time<0.001, Cohen's *f*=1.18; *p*-treatment=0.553. **(c)** Representative photomicrographs of coronal sections of the hypothalamic arcuate nucleus (ARH) from CR control mice treated with vehicle or CNO, showing chromogenic immunohistochemistry for c-Fos (cyan) and fluorescent immunohistochemistry for AgRP (red). Colocalization is indicated by white arrowheads. Scale bars: 50 µm (low magnification) and 25 µm (high magnification). **(d)** Bar graph showing percent body weight change on the fifth day of CR in control (WT: grey; AgRP-Cre: yellow; Gi: purple) and AgRP-Gi mice treated with vehicle (n=12 and 11 per group) or CNO (n=15 and 9 per group). Two-way ANOVA: *p*-treatment × genotype=0.554; *p*-treatment=0.311; *p*-genotype=0.331. Data are presented as mean±SEM; error bars represent SEM.
