# Supplementary material for: AgRP neuron activity enhances reward-related consummatory behaviors during energy deficit in mice
Source: Commun Biol. 2025 Aug 4;8:1152. doi: 10.1038/s42003-025-08620-9 (PMC12321993; doi:10.1038/s42003-025-08620-9)
Supplement: Supplementary file 5 — Reporting Summary [file 42003_2025_8620_MOESM5_ESM.pdf]

nature portfolio

Corresponding author(s): DBPR - COMMSBIO-25-1544

Last updated by author(s): May 27, 2025

Reporting Summary

Nature Portfolio wishes to improve the reproducibility of the work that we publish. This form provides structure for consistency and transparency in reporting. For further information on Nature Portfolio policies, see our Editorial Policies and the Editorial Policy Checklist.

Statistics

For all statistical analyses, confirm that the following items are present in the figure legend, table legend, main text, or Methods section.

n/a	Confirmed	
☐	☒	The exact sample size (*n*) for each experimental group/condition, given as a discrete number and unit of measurement
☐	☒	A statement on whether measurements were taken from distinct samples or whether the same sample was measured repeatedly
☐	☒	The statistical test(s) used AND whether they are one- or two-sided<br>*Only common tests should be described solely by name; describe more complex techniques in the Methods section.*
☐	☒	A description of all covariates tested
☐	☒	A description of any assumptions or corrections, such as tests of normality and adjustment for multiple comparisons
☐	☒	A full description of the statistical parameters including central tendency (e.g. means) or other basic estimates (e.g. regression coefficient) AND variation (e.g. standard deviation) or associated estimates of uncertainty (e.g. confidence intervals)
☐	☒	For null hypothesis testing, the test statistic (e.g. *F*, *t*, *r*) with confidence intervals, effect sizes, degrees of freedom and *P* value noted<br>*Give P values as exact values whenever suitable.*
☒	☐	For Bayesian analysis, information on the choice of priors and Markov chain Monte Carlo settings
☐	☒	For hierarchical and complex designs, identification of the appropriate level for tests and full reporting of outcomes
☐	☒	Estimates of effect sizes (e.g. Cohen's *d*, Pearson's *r*), indicating how they were calculated

Our web collection on statistics for biologists contains articles on many of the points above.

Software and code

Policy information about availability of computer code

Data collection	For data collection to study AgRP remodeling, a Fiji macro was used for quantifications, and it is available in the Zenodo Repository (https://doi.org/10.5281/zenodo.3541615)
Data analysis	For image analysis ImageJ 1.54K was used<br>For statistic analysis GraphPad 8.0 was used

For manuscripts utilizing custom algorithms or software that are central to the research but not yet described in published literature, software must be made available to editors and reviewers. We strongly encourage code deposition in a community repository (e.g. GitHub). See the Nature Portfolio guidelines for submitting code & software for further information.

Data

Policy information about availability of data

All manuscripts must include a data availability statement. This statement should provide the following information, where applicable:

- Accession codes, unique identifiers, or web links for publicly available datasets
- A description of any restrictions on data availability
- For clinical datasets or third party data, please ensure that the statement adheres to our policy

Data will be made available on request.

Research involving human participants, their data, or biological material

Policy information about studies with human participants or human data. See also policy information about sex, gender (identity/presentation), and sexual orientation and race, ethnicity and racism.

Reporting on sex and gender	*Use the terms sex (biological attribute) and gender (shaped by social and cultural circumstances) carefully in order to avoid confusing both terms. Indicate if findings apply to only one sex or gender; describe whether sex and gender were considered in study design; whether sex and/or gender was determined based on self-reporting or assigned and methods used. Provide in the source data disaggregated sex and gender data, where this information has been collected, and if consent has been obtained for sharing of individual-level data; provide overall numbers in this Reporting Summary. Please state if this information has not been collected. Report sex- and gender-based analyses where performed, justify reasons for lack of sex- and gender-based analysis.*
Reporting on race, ethnicity, or other socially relevant groupings	*Please specify the socially constructed or socially relevant categorization variable(s) used in your manuscript and explain why they were used. Please note that such variables should not be used as proxies for other socially constructed/relevant variables (for example, race or ethnicity should not be used as a proxy for socioeconomic status). Provide clear definitions of the relevant terms used, how they were provided (by the participants/respondents, the researchers, or third parties), and the method(s) used to classify people into the different categories (e.g. self-report, census or administrative data, social media data, etc.) Please provide details about how you controlled for confounding variables in your analyses.*
Population characteristics	*Describe the covariate-relevant population characteristics of the human research participants (e.g. age, genotypic information, past and current diagnosis and treatment categories). If you filled out the behavioural & social sciences study design questions and have nothing to add here, write "See above."*
Recruitment	*Describe how participants were recruited. Outline any potential self-selection bias or other biases that may be present and how these are likely to impact results.*
Ethics oversight	*Identify the organization(s) that approved the study protocol.*

Note that full information on the approval of the study protocol must also be provided in the manuscript.

Field-specific reporting

Please select the one below that is the best fit for your research. If you are not sure, read the appropriate sections before making your selection.

☒ Life sciences ☐ Behavioural & social sciences ☐ Ecological, evolutionary & environmental sciences

For a reference copy of the document with all sections, see nature.com/documents/nr-reporting-summary-flat.pdf

Life sciences study design

All studies must disclose on these points even when the disclosure is negative.

Sample size	For studies involving neuroanatomy, a typical sample size often includes around 8 mice per experimental group to ensure reliable results. This number is commonly used in various animal studies to balance statistical power with ethical considerations. In behavior studies, however, it is frequently recommended to use at least 10 mice per group to ensure that the results are robust and can account for individual variability in response to experimental conditions. This sample size helps in providing a higher confidence in the outcomes, particularly when measuring complex behavioral traits.
Data exclusions	For data with a normal distribution, observations exceeding two standard deviations were excluded.
Replication	All the results presented in this manuscript are replications of at least two independent experiments.
Randomization	For randomization, saccharin bottle placement was distributed in such a way that, within identical experimental groups, saccharin was randomly assigned to both sides.
Blinding	For AgRP analysis and c-Fos quantification, the analyses were performed in a blinded manner, without knowledge of the treatment or condition of the sample. The experimental group condition was revealed only for the statistical analysis.

Reporting for specific materials, systems and methods

We require information from authors about some types of materials, experimental systems and methods used in many studies. Here, indicate whether each material, system or method listed is relevant to your study. If you are not sure if a list item applies to your research, read the appropriate section before selecting a response.

Materials & experimental systems

n/a	Involved in the study
☐	☒ Antibodies
☒	☐ Eukaryotic cell lines
☒	☐ Palaeontology and archaeology
☐	☒ Animals and other organisms
☒	☐ Clinical data
☒	☐ Dual use research of concern
☒	☐ Plants

Methods

n/a	Involved in the study
☒	☐ ChIP-seq
☒	☐ Flow cytometry
☒	☐ MRI-based neuroimaging

Antibodies

Antibodies used

anti-cFos antibody (Abcam cat. ab214672; RRID:AB_2939046) (Synaptic Systems, 226308; RRID: AB_2905595)
anti-AgRP antibody (Phoenix Pharmaceuticals cat. H-003-57; RRID:AB_2313909)
anti-hrGFP (Stratagene cat. 240142; RRID:AB_2314658)
anti-rabbit antibody Alexa Fluor 594 (Thermo Fisher Scientific cat. A-21207; RRID: AB_141637)
anti-rabbit antibody Alexa Fluor 488 (Thermo Fisher Scientific cat. A-32790; RRID:AB_2762833)
anti-guinea pig antibody Alexa Fluor 488 (Thermo Fisher Scientific, A-11073, RRID:AB_2534117)
biotinylated anti-rabbit antibody (VectorLabs; RRID:AB_2313606)

Validation

anti-cFos antibody (Abcam cat. ab214672; RRID:AB_2939046) -> rabbit monoclonal antibody suitable for human and mouse samples.

anti-cFos antibody (Synaptic Systems, 226308; RRID: AB_290559) -> This antibody is a chimeric antibody based on the monoclonal rat antibody clone 108B5. The constant regions of the heavy and light chains have been replaced by Guinea pig specific sequences. Therefore, the antibody can be used with standard anti-Guinea pig secondary reagents. Reacts with: mouse (P01101), rat (P12841), human (P01100).

anti-AgRP antibody (Phoenix Pharmaceuticals cat. H-003-57; RRID:AB_2313909) -> rabbit polyclonal antibody suitable for human and mouse

anti-hrGFP (Stratagene cat. 240142; RRID:AB_2314658) -> rabbit polyclonal antibody. it was use in many publications (https://pmc.ncbi.nlm.nih.gov/articles/PMC4604734/ ; https://pmc.ncbi.nlm.nih.gov/articles/PMC2697121/ ; https://www.nature.com/articles/s41467-020-14498-4) and in a patent (https://patents.google.com/patent/US8404658B2/en)

Animals and other research organisms

Policy information about studies involving animals; ARRIVE guidelines recommended for reporting animal research, and Sex and Gender in Research

Laboratory animals

Mouse C57BL6J. 2- to 5-month-old male

Wild animals

The study did not involve wild animals

Reporting on sex

In this study, we exclusively used male subjects to minimize the potential confounding effects of hormonal fluctuations associated with the estrous cycle in females. Given that our experimental design focuses on the role of AgRP neurons signaling under caloric restriction, we aimed to reduce variability that could arise from sex-specific differences in metabolic and behavioral responses. There are many evidences that revealed several sexually differentiated responses to metabolic challenges in C57BL/6 mice, highlighting the importance of taking into account sex differences in metabolic studies. Future studies should explore whether similar effects are observed in females to determine the extent to which these findings generalize across sexes

Field-collected samples

The study did not involve samples collected from the field

Ethics oversight

All experiments were approved by the Institutional Animal Care and Use Committee of the IMBICE or Biophysics Department of UNIFESP

Note that full information on the approval of the study protocol must also be provided in the manuscript.

Plants

Seed stocks

Report on the source of all seed stocks or other plant material used. If applicable, state the seed stock centre and catalogue number. If plant specimens were collected from the field, describe the collection location, date and sampling procedures.

Novel plant genotypes

Describe the methods by which all novel plant genotypes were produced. This includes those generated by transgenic approaches, gene editing, chemical/radiation-based mutagenesis and hybridization. For transgenic lines, describe the transformation method, the number of independent lines analyzed and the generation upon which experiments were performed. For gene-edited lines, describe the editor used, the endogenous sequence targeted for editing, the targeting guide RNA sequence (if applicable) and how the editor was applied.

Authentication

Describe any authentication procedures for each seed stock used or novel genotype generated. Describe any experiments used to assess the effect of a mutation and, where applicable, how potential secondary effects (e.g. second site T-DNA insertions, mosiacism, off-target gene editing) were examined.
